# Elastomeric Core/Conductive Sheath Fibers for Tensile and Torsional Strain Sensors

**DOI:** 10.3390/s22228934

**Published:** 2022-11-18

**Authors:** Jeeeun Kim, Changsoon Choi

**Affiliations:** Department of Energy and Materials Engineering, Dongguk University, Seoul 04620, Republic of Korea

**Keywords:** carbon nanotubes, polymer composite fiber, strain sensor, multi-mode, wearable sensor

## Abstract

Motion sensing, aimed at detecting and monitoring mechanical deformation, has received significant attention in various industrial and research fields. In particular, fiber-structured mechanical strain sensors with carbon-based materials have emerged as promising alternatives for wearable applications owing to their wearability and adaptability to the human body. Various materials, structures, sensing mechanisms, and fabrication methods have been used to fabricate high-performance fiber strain sensors. Nevertheless, developing multi-modal strain sensors that can monitor multiple deformations remains to be accomplished. This study established core/sheath fiber multi-modal strain sensors using polymer and carbon nanotubes (CNTs). Specifically, a flexible and conductive CNT sheet was wrapped onto the elastomeric core fiber at a certain angle. This wrapping angle allowed the CNTs to mechanically deform under tensile and torsional deformations without fatal structural damage. The CNTs could sense both tensile and torsional strains through reversible structural changes during deformations. The fiber strain sensor exhibited an increase of 124.9% and 9.6% in the resistance during tensile and torsional deformations of 100% and 1250 rad/m, respectively.

## 1. Introduction

Motion sensing is an actively studied research topic that aims to detect and monitor the position, movement, and mechanical deformation [1,2,3,4,5]. In the field of wearable devices, motion sensing has attracted considerable attention because human motion contains enormous amounts of information related to their physical conditions and health situation [6,7,8,9,10,11], and this information can form the basis for various applications related to health and medical care [5,12,13,14]. Fiber-structured mechanical strain sensors, which are compact, low weight, and mechanically flexible, are the most promising sensors for wearable devices owing to their wearability and adaptability to the human body [8,15,16].

Several researchers have mainly employed two fiber structures to realize fiber strain sensors. Fiber sensors with core/sheath structure in which an elastomeric core substrate fiber [4,16,17,18,19,20,21,22,23] is wrapped with flexible and electrically conductive carbon-based nanomaterials (such as carbon black, carbon nanotubes (CNTs), and graphene) are simple and easy to fabricated [1,7,24,25]. Other researchers produced coiled structures with carbon material-based fibers and realized mechanical strain sensing through increased resistance induced by changes in the contact area between the adjustable coil loops during deformation [26,27,28,29,30,31]. Unfortunately, existing fiber strain sensors share a critical limitation of the inability to sense complex deformations. Human motion generates various mechanical deformations, including bending, stretching, and twisting, but most previously reported fiber strain sensors have been devoted toward single-modal sensing. Different materials, structures, sensing mechanisms, and fabrication methods for sensing each type of deformation [11,12,32,33,34,35,36] impede the integration and establishment of multi-modal wearable platforms, which have a minimal surface and volume. Although several pioneering researchers have reported multi-modal fiber strain sensors [2,34,35,37,38,39], the complex structure and low productivity still require reasonable and innovative structural designs [4,39,40].

In this study, we developed a single fiber strain that can sense both tensile and torsional strains through standard strain components. The fiber strain sensor was fabricated by wrapping a flexible and electrically conductive well-aligned CNT sheet on a stretchable core substrate fiber with a specific wrapping angle. When mechanical deformation was applied, the shear strain in the perpendicular direction led to macroscopic changes in the wrapping angle, thereby providing mechanical deformability to the CNT sheet. The microscopic reversible structural changes in the CNT sheet induced by the normal strain along the longitudinal direction allowed the fiber to sense mechanical strains with increased resistance. Consequently, the fiber strain sensor exhibited changes of 124.9% and 9.6% in the resistance during tensile and torsional deformations up to 100% and 1250 rad/m, respectively. The proposed fiber strain sensors can be applied in wearable devices and various high-tech industrial and research applications, such as smart clothing, medical care, IoT, and robots.

## 2. Results

### 2.1. Fabrication of the CNT-Wrapped Core/Sheath Fiber Strain Sensors

**Figure 1:**
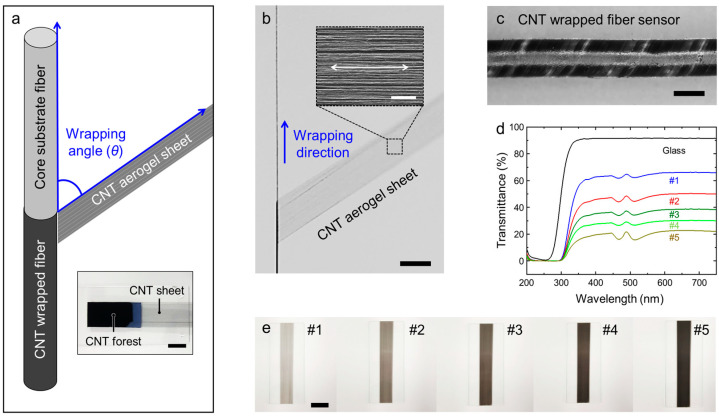
Schematic and fabrication of the CNT-wrapped core/sheath fiber strain sensor. (**a**) Schematic showing the CNT being wrapped on the core substrate fiber with a specific wrapping angle (θ). (Inset: photograph of the forest-drawn CNT aerogel sheet, scale bar = 5 mm). Photograph showing (**b**) the CNT being wrapped on the core substrate fiber, with the white arrow indicating the CNT alignment direction (scale bar = 1 cm) (Inset: scanning electron microscopy (SEM) image of forest-drawn well-aligned CNT aerogel sheet, scale bar = 50 μm) and (**c**) CNT-wrapped fiber strain sensor with the wrapping angle on the surface. (**d**) Optical transmittance versus wavelength of stacked CNT sheet with one to five layers. (**e**) Photographs showing the dependence of the transparency on the number of layers in the stacked CNT sheet (scale bar = 1 cm).

Figure 1a schematically illustrates the core/sheath fiber strain sensor. The fiber sensor comprises an elastomeric core fiber substrate and a conductive sheath. Elastomeric fibers with a diameter of 1360 μm are prepared using commercially available ecoflex 0030 and used as a core substrate fiber. Such silicone rubbers are commercially available, stretchable, easy to manufacture without limitation in fiber length. A polymer solution for elastomeric core fiber was prepared by mixing the subject and curing agents with a weight ratio of 1:1. After bubble elimination in a vacuum chamber, the mixture was filled in a 16 G size syringe and cured in an oven at 80 ℃ for 2 h. The finished product can be fully stretched in the tensile direction of 400%. 

Multi-walled carbon nanotube (CNT) aerogel sheets drawn from the CNT forest has a unique structure of well-aligned bundles [41]. Thanks to this structure, CNT sheets exhibited high mechanical and electrical properties; the anisotropic structure reduces the performance degradations by minimizing the physical defects between CNT nanobundles. In addition, the millions of nanobundles and mesoporous inter-bundle gaps allow close contact and strongly interact with external materials, such as various polymers, without additional binders. The 1 cm wide CNT sheets are drawn from a vertically standing CNT forest grown by chemical vapor deposition (CVD) with a typical height of 290 μm (inset of Figure 1a). The CNT sheets are wrapped around a core fiber with a specific wrapping angle using customized twisting machines. The wrapping angle allows the CNTs, which are mechanically flexible and electrically conductive but inelastic, to mechanically deform. As the wrapping process continues, the CNT aerogel sheets cover the fiber along the longitudinal direction of the core substrate fiber (Figure 1b).

The scanning electron microscopy (SEM) image shows the alignment of bundles in the CNT aerogel sheet, which was maintained even after the wrapping process (inset of Figure 1b). Subsequently, a small amount of ethanol was drop-casted to the CNT-wrapped core/sheath fiber to densify the CNT sheet through the surface-tension-based densification and prevent the detachment of CNTs from the surface of the core fiber. Figure 1c, which is the optical photograph of the resulting CNT-wrapped core/sheath fiber, clearly shows the wrapping angle on the fiber surface. Figure 1d shows the relationships between the number of stacked CNT layers and transmittance as a function of the wavelength. As the number of CNT layers increases from one to five, the transmittance in the wavelength range between 300 and 750 nm decreases from 63% to 19% (the transmittance of slide glass is approximately 91%). These optical properties can also be observed in the optical photographs, in which the CNTs turn deep black as the number of CNT layers increases (Figure 1e).

### 2.2. Experimental Setup for Resistance Measurement and Morphology of the CNT-Wrapped Core/Sheath Fiber Strain Sensor

**Figure 2:**
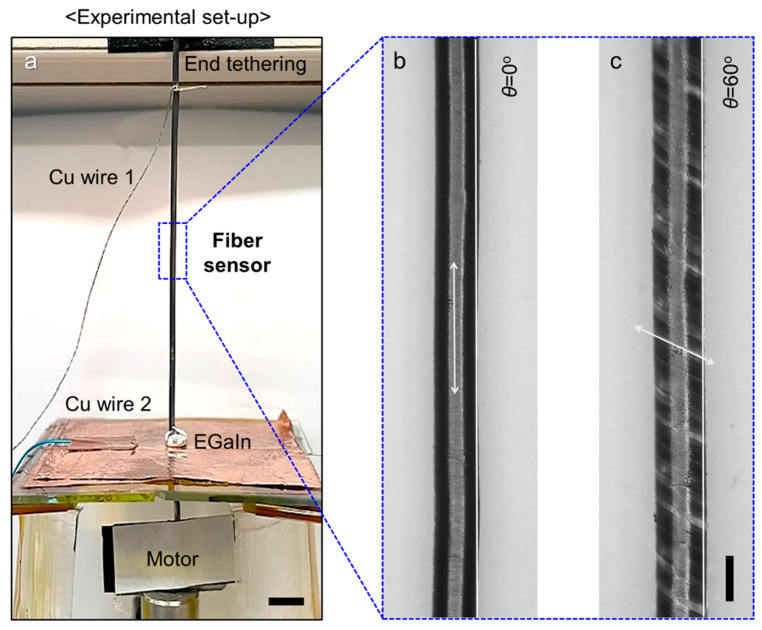
Measurement setup and morphology of the CNT-wrapped core/sheath fiber strain sensor. (**a**) Photograph of the experimental setup for resistance measurement during tensile and torsional deformation (scale bar = 5 mm). Magnified photographs showing CNT-wrapped fiber sensors with (**b**) zero wrapping angle (θ = $0^{{}^{\circ}}$) and (**c**) nonzero wrapping angle (θ = $60^{{}^{\circ}}$) (scale bar = 1 mm).

Figure 2a illustrates the experimental setup for resistance measurement during mechanical deformations. The setup is composed of a CNT-wrapped core/sheath fiber strain sensor, a rotational motor, and electrically conductive Cu electrodes. The upper end of the fiber strain sensor is vertically hung with the end tethering, and the opposite lower end is attached to the motor tip for applying torsional deformation. Both ends of the fiber strain sensors are connected to the conductive electrodes. In particular, the lower end is passed through the center holes in the conductive Cu plate and electrically connected with a droplet of eutectic gallium indium (EGaIn) liquid metal alloy to minimize the contact resistance and physical effects of the attached electrode during torsional deformation. Two types of fibers with different wrapping angles, zero (θ = $0^{{}^{\circ}}$, Figure 2b) and nonzero (θ = $60^{{}^{\circ}}$, Figure 2c), are fabricated. The morphological differences in the fiber strain sensors with wrapping angles of $0^{{}^{\circ}}$ and $60^{{}^{\circ}}$ can be clearly observed in the optical photographs (Figure 2b,c, respectively). The dependence of the resistance on the tensile deformations is measured for tensile strain ranging from 0% to 100% by changing the distance from the upper end and lower end fixed to the motor tip. The torsional-deformation-dependent changes in the resistance are measured by applying torsional deformation between 0 and 1250 rad/m. The upper end of the fiber strain sensor is tethered to prevent unintended rotation during torsional deformation.

### 2.3. Electrical and Morphological Changes of the CNT-Wrapped Core/Sheath Fiber Tensile Strain Sensors

**Figure 3:**
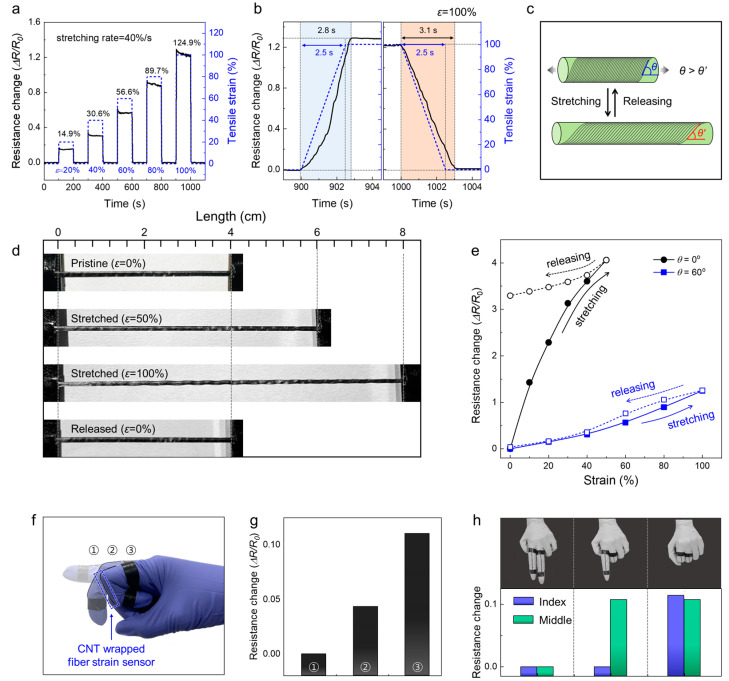
Electrical performances and morphological changes in CNT-wrapped core/sheath fiber strain sensor during tensile deformation. (**a**) Dynamic response curves of fiber sensors under various tensile strains (20%, 40%, 60%, 80%, 100%). (**b**) Response and recovery time of CNT-wrapped fiber strain sensor during stretching 100% and releasing to initial state. (**c**) Schematic showing the changes in the reversible CNT wrapping angle during the stretching and releasing processes. (**d**) Photographs showing the fiber in the pristine state, 50% tensile strain, 100% tensile strain, and pristine state after release. (**e**) Resistance changes versus tensile strain (from 0% to 100%) of CNT-wrapped core/sheath fiber strain-sensing fiber sensors with zero wrapping angle (θ = $0^{{}^{\circ}}$) and nonzero wrapping angle (θ = $60^{{}^{\circ}}$). ΔR is the change in the fiber resistance under tensile deformation, and $R_{0}$ is the fiber resistance in the initial state. (**f**) Photograph and (**g**) response of the 5 cm long CNT-wrapped fiber strain sensor attached to the index finger with various hand motions. (**h**) Photographs of sensors attached to the index and middle fingers and resistance changes during various hand gestures.

The CNTs wrapped around the core substrate fiber enable the sensing of the tensile and torsional strains. The dynamic response curves of the CNT-wrapped fiber sensors were obtained under various tensile strains (Figure 3a). As the tensile strain increased from 20% to 100% (tensile deformation rate = 40%/s), the resistance of fiber sensors progressively increased from 14.9% to 124.9%. The tensile strains were held for 100 s and released to the initial state with the same deformation rate. As shown in Figure 3b, our fiber sensors exhibited 2.8 s (to the highest value) and 3.1 s (to the lowest value), respectively, in the enlarged graphs of resistance changes during 2.5 s of 100% tensile strain and releasing. Figure 3c schematically illustrates the simplified structural model of the fiber strain sensor with a specific wrapping angle before and after the application of tensile deformation. Owing to the macroscopic changes in the structural angle of the wrapped CNTs, the CNTs, which are flexible but inelastic, can mechanically deform, generating a tensile strain of up to 100% with reversible recovery (Figure 3d). In addition, this tensile strain decreases the wrapping angle and increases the spacing between the CNT bundles. In the pristine state, the electrons are transported along the aligned CNT bundles, i.e., the direct conductive pathway. Furthermore, the increased bundle spaces, which can also be interpreted as slight structural damages, induce indirect electron transport and result in extended pathways. 

The resistance changes ($\Delta R/R_{0}$) of CNT-wrapped core/sheath fiber strain sensors with wrapping angles of $0^{{}^{\circ}}$ and $60^{{}^{\circ}}$ are compared in Figure 3e. The resistance change is the ratio of the difference in the resistance of the fiber strain sensor before and after applying mechanical deformations (ΔR) by the resistance in the pristine state ($R_{0}$). The fiber strain sensor with a wrapping angle of $60^{{}^{\circ}}$ exhibits an increase of approximately 124.9% in the resistance during 100% tensile strain, and the increased resistance recovers to the initial value after the sensor is released to the pristine state with negligible changes in the initial resistances before and after deformation.

As shown in Figure S1a,b, the resistance changes are well maintained during 100 cycles of repeated tensile deformation (103% resistance retention). In contrast, a drastic increase in the resistance is measured at the 100% tensile strain (approximately a fourfold increase) for the fiber strain sensor with a wrapping angle of $0^{{}^{\circ}}$. Moreover, hysteresis occurs, so the increased resistance does not recover to the initial value even after release. The resistance remains in the increased states with repeated tensile deformation cycles (Figure S2). The morphological characterization and strain sensing mechanism are presented to clarify the mechanical and electrical performance changes in the fiber strain sensors. The tensile deformation induces a fatal destructive effect on the CNT sheet with a wrapping angle of $0^{{}^{\circ}}$ and results in bundle breakages because the CNT bundles along the longitudinal direction of the fiber strain sensor cannot effectively absorb the mechanical strain during deformation (Figure S3). These material destructions are permanent, and damages remain even after release, causing the CNT electrode to lose its intrinsic electrical conductivity. Accordingly, although the sensitivity of the fiber strain sensor with a wrapping angle of $60^{{}^{\circ}}$ is low, it can be used repeatedly within the reversible tensile deformation range. The detailed sensing mechanism is described in the last section of the results and discussion. The key performance parameters of our strain sensors (structures, materials, types of deformation, sensitivity, and sensing range) were compared to those of previously reported fiber-type strain sensors (Table S1).

Further experiments were conducted to investigate the effects of environmental parameters on sensing performances. The resistance changes of CNT-wrapped fiber strain sensors with a wrapping angle of $60^{{}^{\circ}}$ were measured in various temperatures of 5, 25, and 45 ℃, where the fiber sensors were placed in each temperature environment for about an hour. Regardless of environmental temperatures, fiber sensors could sense the tensile deformations (Figure S4a); the difference in sensing performance was only 5.7%, corresponding to a temperature increase from 5 to 45 ℃. In addition, the effect of environmental humidity under various relative humidity (RH) conditions of 20, 50, and 80 RH% was also investigated. The sensing performance of sensors was scarcely affected, with negligible differences (Figure S4b).

We demonstrated the potential application of our CNT-wrapped fiber strain sensors for human motion monitoring sensors. As shown in Figure 3f, the 5 cm long fiber strain sensor was attached to the index finger and electrically connected to an external multi-meter with copper wire. As the index finger was bent, the fiber sensor was stretched and induced the changes in resistance. As the finger was bent progressively, the resistance was increased by 4.3% and 11.1%, respectively (Figure 3g). Furthermore, two fiber sensors attached to the index and middle fingers were employed to distinguish various hand motions (Figure 3h). Each fiber sensor exhibited different resistance changes along with various hand gestures. 

### 2.4. Electrical and Morphological Changes of the CNT-Wrapped Core/Sheath Fiber Torsional Strain Sensors

**Figure 4:**
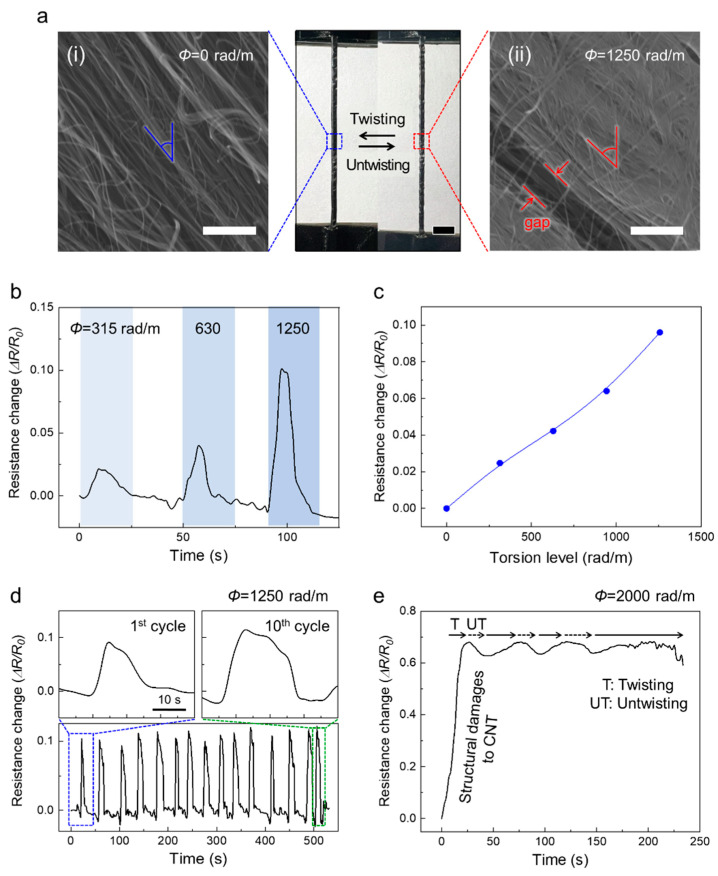
Electrical performances and morphological changes of CNT-wrapped core/sheath fiber strain sensor during torsional deformation. (**a**) Photographs of CNT-wrapped core/sheath fiber strain sensors with nonzero wrapping angle (θ = $60^{{}^{\circ}}$) before and after applying torsional deformation of 1500 rad/m. Magnified SEM images of (**i**) well-aligned CNT bundles of fiber sensor in the pristine state and (**ii**) damaged CNT bundles after applying torsional deformation. (**b**) Change in the resistance of fiber sensors under various levels of torsional deformations (315, 630, and 1250 rad/m). Resistance changes (**c**) versus torsional strains from 0 to 1250 rad/m and (**d**) during 10 cycles of torsion deformations of 1250 rad/m. Magnified graphs showing the resistance changes in the 1st and 10th cycles. (**e**) Irreversible changes in the resistance of the fiber sensor under excessive torsional deformation. The CNT sheet undergoes permanent structural damage during deformation.

The morphological and electrical changes under torsional deformation are compared. The center part of Figure 4a shows the optical photographs of the fiber strain sensor with a wrapping angle of $60^{{}^{\circ}}$ before and after the application of torsion deformation of 1250 rad/m. Macroscopically, the wrapping angle and fiber length slightly increase during the full deformation range. As the change in the wrapping angle endows tensile deformability to the CNTs, the macroscopic structural angle changes help the fibers absorb torsional strain, providing the CNTs with a certain torsional tolerance and allowing them to reversibly twist and untwist. The surface SEM images of the fiber sensor show the microscopic structure changes (left and right parts of Figure 4a). Unlike the well-aligned CNT sheets in the fiber strain sensor in the pristine state, islands, bundle gaps, and inter-fibrils are observed in the sheet torn by torsional deformation. The inter-fibrils connecting the island segments enlarge the electrically conductive pathway in the longitudinal direction of the fiber strain sensor, resulting in increased resistance. This structural damage, which is also observed during the tensile deformations, allows the monitoring of both mechanical strains (tensile and torsional) through changes in the electrical resistances. Figure 4b shows the change in the resistance of the fiber strain sensor with a wrapping angle of $60^{{}^{\circ}}$ under torsional deformations of 315, 630, and 1250 rad/m. The fiber strain sensor exhibits an increase of 2%, 4%, and 9.6% in the resistance, and the increased values reversibly recover to the initial values after untwisting to the pristine states. Figure 4c illustrates the dependence of the resistance changes on the torsional strain. The torsional strain levels and resistance changes exhibit a linearly proportional relationship in the strain range of 0 to 1250 rad/m. As shown in Figure 4d, the fiber sensors can withstand several torsional deformation cycles and exhibit reasonable stability. The resistance changes are well maintained and recover reversibly during 10 repeated deformation cycles of 1250 rad/m. The graphs at the top of Figure 4d present a comparison of the changes in the resistance of the first and last cycles. The reversibility and stability of the fiber sensor imply that the structural destruction of the CNTs by the torsional deformation is insignificant and temporary. However, a drastic and irreversible increase in the resistance is observed beyond the deformation of 1250 rad/m; increased resistance is observed even after untwisting to the initial state (Figure 4e). Similar to the structural damages of the fiber sensor with a wrapping angle of $0^{{}^{\circ}}$ after tensile deformation, the CNT alignment is observed to be disrupted in the optical photograph (Figure S5).

### 2.5. Deformation Sensing Mechanism of the CNT-Wrapped Core/Sheath Fiber Strain Sensors

**Figure 5:**
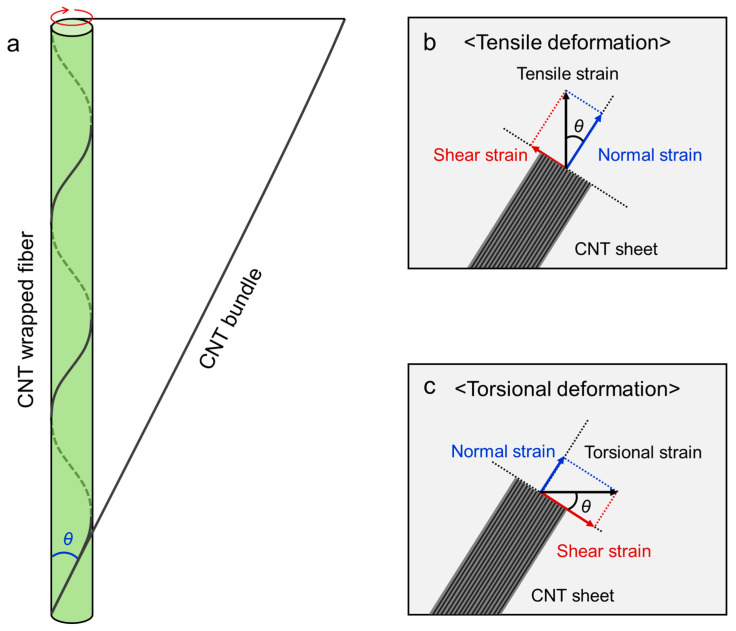
Tensile and torsional deformation sensing mechanism of CNT-wrapped core/sheath fiber strain sensor. (**a**) Schematic showing helical spring model for core/sheath CNT-wrapped fiber strain sensor with wrapping angle θ. Vector decomposition of (**b**) tensile and (**c**) torsional strain vectors applied to the CNT sheet with wrapping angle θ. Blue and red arrows indicate normal and shear strain vectors, respectively.

Figure 5a shows a helical spring model of the CNT-wrapped core/sheath fiber strain sensor with a wrapping angle of θ. Well-aligned CNT bundles drawn from the forest are helically wrapped on the core substrate fiber with a predetermined wrapping angle during fabrication. The wound bundle can be transformed into a straight bundle by unwinding from the core fiber, representing the physical relationship among the CNT-wrapped fiber strain sensor, CNT bundle, and wrapping angle. This model allows the vector analysis of the mechanical strains applied to the CNT sheet during tensile and torsional deformations. As shown in Figure 5b,c, the mechanical strains can be decomposed into normal and shear strains, implying that the CNT sheets undergo longitudinal and perpendicular strains along the length direction under both tensile and torsional deformations (the blue and red arrows represent the normal strain for the longitudinal component and shear strain for the perpendicular component, respectively). The magnitudes of each vector component depend on the wrapping angles. The shear strain affects the macroscopic predetermined wrapping angles and endows the CNT-wrapped fiber strain sensors with mechanical deformability and tolerance. The effects of the shear component are supported by changes in wrapping angle expressed as the following equation [42]:

(1) tanθ=2πrT,

where θ is the wrapping angle, *r* is the diameter of the fiber, and *T* is the inserted twist in turns per meter of the fiber length. In the case of tensile deformation, a shear strain, which is applied in the CNT wrapping direction, decreases the wrapping angle and provides a tensile tolerance of reversible stretching and releasing (Figure 3c). Contrary to tensile deformation, the wrapping angle increased due to the shear strain applied in the unwrapping direction during torsional deformation (Figure S6). 

Moreover, the normal strains of both deformations cause microscopic fractures throughout the CNT sheet and create islands, bundle gaps, and inter-fibrils. The inter-fibrils connecting the islands lengthen the electrically conductive pathway in the fiber direction, resulting the increases in resistance. In contrast, when tensile deformation is applied to the CNT fiber strain sensor with a wrapping angle of $0^{{}^{\circ}}$ or excessive mechanical deformations are applied, the highest normal strain permanently damages the CNT sheets, leading to irreversible resistance changes that hinder repeated uses (Figure 3a and Figure 4e). In this perspective, the sensitivity and sensing range of the fiber strain sensor are in a trade-off relationship, and a specific wrapping angle that can reduce the normal destructive strain and contribute to a wide sensing range and repeatability instead of low sensitivity is preferred.

## 3. Conclusions

A CNT/polymer composited multi-modal fiber motion sensor that can monitor both tensile and torsional deformations was demonstrated. The proposed fiber sensor was fabricated by wrapping a mechanically flexible and electrically conductive CNT sheet on the stretchable core fiber with a specific wrapping angle. The CNTs can sense mechanical deformations through microscopic structural changes, and the changes in the wrapping angle allow the CNTs to deform without fatal structural damage. The resulting fiber sensor exhibited a reversible tensile strain of up to 100%, with an increase of 124.9% in the resistance. Furthermore, the resistance increased by 9.6% during torsional strain up to 1250 rad/m. These findings highlight that the proposed multi-modal fiber strain sensor can be applied in high-tech industries and diverse research fields such as wearable devices, smart clothing, medical care, IoT, and robots.

## Data Availability

Not applicable.

## Supplementary Materials

The following supporting information can be downloaded at: https://www.mdpi.com/article/10.3390/s22228934/s1. Figure S1: (a) Resistance retention of CNT-wrapped fiber strain sensor with a wrapping angle of $60^{{}^{\circ}}$ versus 100 cycles of repeated tensile deformations. (b) Resistance changes of CNT-wrapped fiber strain sensor at 1st and 10th deformation cycles; Figure S2: Change in the resistance of CNT-wrapped fiber strain sensor with a wrapping angle of $0^{{}^{\circ}}$ during repeated tensile deformations; Figure S3: Optical images showing the morphological changes on the surface of the fibers with zero wrapping angle (*θ* = $0^{{}^{\circ}}$) and nonzero wrapping angle (*θ* = $60^{{}^{\circ}}$) at pristine, 100% tensile strain, and pristine state after releasing (scale bar = 1 cm); Figure S4: Resistance changes of sensor under 100% strain in various (a) temperatures and (b) relative humidity (RH) conditions; Figure S5: Optical photograph showing the surface of core/sheath fiber strain sensor with a wrapping angle of $60^{{}^{\circ}}$ after applying and releasing excessive torsional deformation (scale bar = 1 mm); Figure S6: Schematic showing the changes of CNT wrapping angle by torsional deformation. These changes are reversible during the stretching-releasing and twisting-untwisting processes; Table S1: The tensile and torsional performance comparison of previous fiber-type strain sensors and this work.
