# Supplementary material for: Elastomeric Core/Conductive Sheath Fibers for Tensile and Torsional Strain Sensors
Source: Sensors (Basel). 2022 Nov 18;22(22):8934. doi: 10.3390/s22228934 (PMC9693023; doi:10.3390/s22228934)
Supplement: Supplementary file 1 [file sensors-22-08934-s001.zip › sensors-2035810-supplementary.pdf]

Supplementary Information

Elastomeric Core/Conductive Sheath Fibers for Tensile and Torsional Strain Sensors

Jeeeun Kim and Changsoon Choi *

Department of Energy and Materials Engineering, Dongguk University, Seoul 04620, Republic of Korea
* Correspondence: cschoi84@dongguk.edu

Materials and Methods

Preparation of Core Substrate Fiber Commercially available ecoflex 0030 (Smooth-On, Inc) was used as the substrate materials for the elastic core substrate fiber. An ecoflex solution was prepared by mixing 5 g each of the subject and curing agents (weight ratio of 1:1) and put it in vacuum chamber for 5 minutes to eliminate air bubbles. The bubble-less mixture was filled in a 16 G size (diameter of 1360 μm) and 8-cm-long syringe (Korea Vaccine Co. Ltd.) with and cured at room temperature for 6 hours or in an oven at 80 ℃ for 2 hours.

CNT-wrapped fiber sensor First, to fabricate a fiber sensor with a zero wrapping angle, one layer of 1-cm-wide carbon nanotube (CNT) sheet was drawn from well-aligned multi-walled carbon nanotube (MWNT) forests (A-tech system Co., Korea) grown by chemical vapor deposition (CVD) with a typical height of 290 μm and loaded onto the longitudinal direction of core substrate fiber. The CNT sheet was rolled around the core substrate fiber on one side parallel to the CNT sheet, and a small amount of ethanol was dropped onto the fiber for vapor densification to prevent detachment from the core. To fabricate a fiber sensor with a specific wrapping angle, both sides of the core fiber were fixed to the tips of two customized motors (K6G3C, GGM). After loading a 1-cm-wide CNT sheet at one end of the core fiber at a desired winding angle, the fiber was twisted in one direction. The aligned CNT sheet was continuously drawn from the CNT forest as the drawn sheet to be wrapped around the core fiber. Similarly, the CNT fiber wrapped with a specific angle was densified by ethanol.

Characterization Copper wires with a diameter of 180 μm, silver paste, and eutectic gallium indium (EGaIn, Sigma Aldrich) were used to prepare electrical connections. SourceMeter

(Keithley 2450) was used as a resistance monitoring device during tensile and torsional deformation. To evaluate the sensing performances of the tensile and torsion strains, the upper fixed end was connected with copper wire and silver paste, and the lower end was electrically connected using a conductive plate with a hole and liquid metal EGaIn. During the deformation, a constant voltage (5 V) was applied to the fiber sensors to acquire a real-time resistance variation and current signal, using an electrochemical workstation (PARSTAT 2273, Princeton Applied Research).

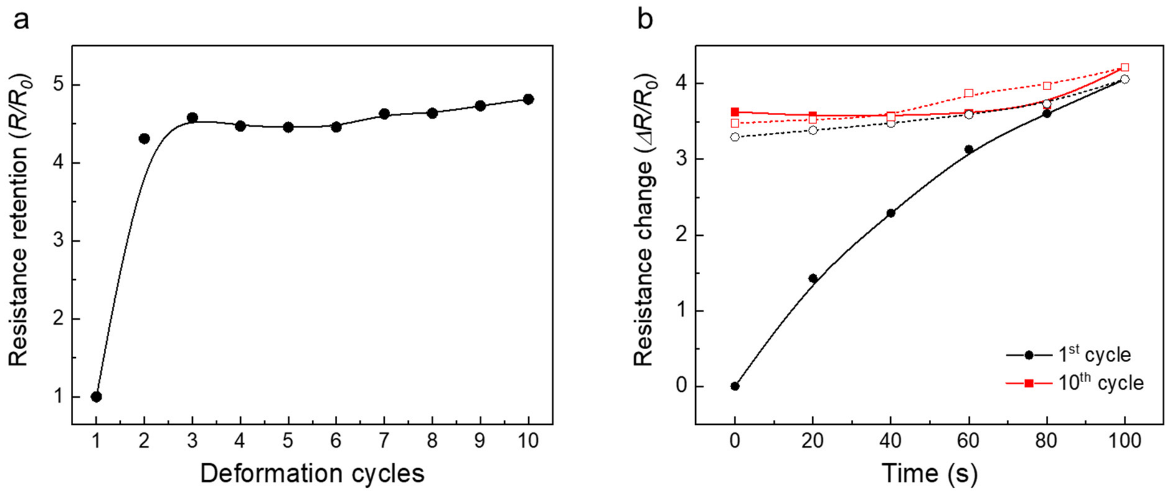

Figure S1. (a) Resistance retention of CNT-wrapped fiber strain sensor with a wrapping angle of 60° versus 100 cycles of repeated tensile deformations. (b) Resistance changes of CNT-wrapped fiber strain sensor at 1st and 10th deformation cycles.

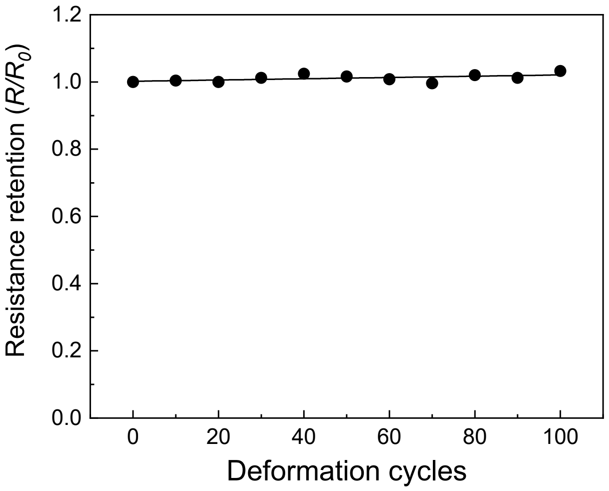

Figure S2. Change in the resistance of CNT-wrapped fiber strain sensor with a wrapping angle of 0° during repeated tensile deformations.

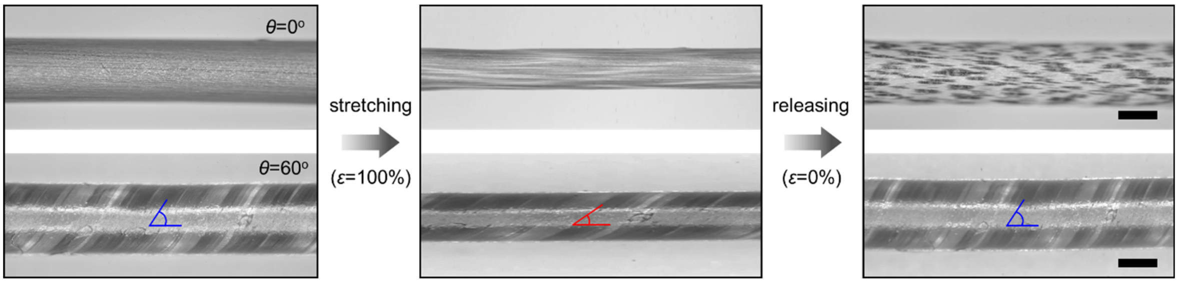

Figure S3. Optical images showing the morphological changes on the surface of the fibers with zero wrapping angle ($\theta = 0^{\circ}$) and nonzero wrapping angle ($\theta = 60^{\circ}$) at pristine, 100% tensile strain, and pristine state after releasing (scale bar = 1 cm).

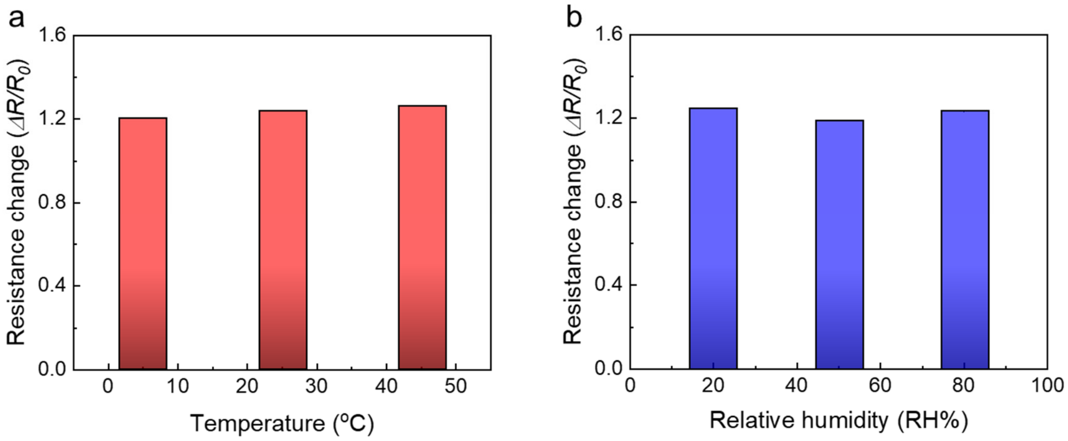

Figure S4. Resistance changes of sensor under 100% strain in various (a) temperatures and (b) relative humidity (RH) conditions.

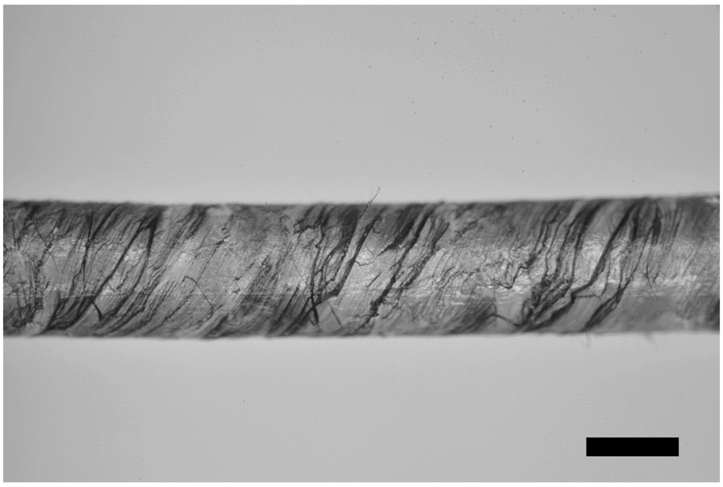

Figure S5. Optical photograph showing the surface of core/sheath fiber strain sensor with a wrapping angle of 60° after applying and releasing excessive torsional deformation (scale bar = 1 mm).

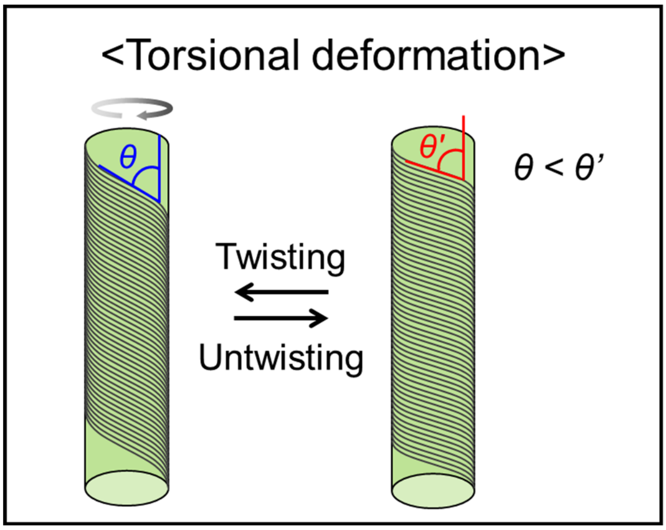

Figure S6. Schematic showing the changes of CNT wrapping angle by torsional deformation. These changes are reversible during the stretching-releasing and twisting-untwisting processes.

Table S1. The tensile and torsional performance comparison of previous fiber-type strain sensors and this work.

	Ref (#)	Structures	Materials	Diameter (mm)	Sensitivity	Sensing range
	**Our work**	**Core/sheath**	**CNT/ecoflex**	**1.36**	**1.25**	**100%<br>1250 rad/m**
**Tensile**	4	Fiber	RGO/PDCY	0.65	3.7	100%
	25	Core/sheath	Silicone elastomer/CNT	1.71	1378	330%
	10	Fiber	CNT/TPU	0.13	2800	100%
	31	Helical yarn	CNT/PU	1.196	-	900%
	22	Fiber	CNT/EVA	-	3.25~33.29	190%
	23	Fibrous tube	CNT/TPU	-	57	760%
**Torsional**	4	Fiber	RGO/PDCY	0.65	2	800 rad/m
	31	Helical yarn	CNT/PU	1.196	-	12.57 rad
	17	Rod	CNT/PDMS	3.0	-	400 rad/m
	18	Rod	Graphene/PDMS	4.0	0.3	800 rad/m
